# Occurrence of multiple fistulas decades after ingestion and neglect of numerous thermometers: a case report

**DOI:** 10.1186/s40792-023-01801-w

**Published:** 2024-01-02

**Authors:** Katrin Schulte, Henning Wendelin Wolf

**Affiliations:** General and Visceral Surgery, Cantonal Hospital Aarau, Tellstrasse 25, 5001 Aarau, Switzerland

**Keywords:** Foreign body ingestion, Thermometer, Endoscopic removal, Fistulas, Case report

## Abstract

**Background:**

Ingestion of thermometers is a very rare occurrence and associated with penetrations of hollow organs. An event decades ago can lead to the development of fistulas.

**Case presentation:**

We present a case of a 62-year-old male who swallowed multiple thermometers with a length of up to 22 cm over a period of 40 years. Diagnostic imaging presented a retroperitoneal abscess due to a duodenal perforation of the longest thermometer as well as multiple other thermometers stuck in the small intestine. After all thermometers were removed and the abscess drained, the patient showed a clinical deterioration. In further operations we found a duodeno-sigmoid fistula and a gastro-thoracal fistula, which were not visible in the initial operations and imaging.

**Conclusion:**

We recommend an active search for fistulas especially in the case of long-foregone ingestion.

**Supplementary Information:**

The online version contains supplementary material available at 10.1186/s40792-023-01801-w.

## Background

Swallowing foreign bodies such as toys or coins is common in children but occurs less in adults. Objects such as bones are usually accidentally swallowed with food, whereas intentional swallowing of larger foreign bodies is more common in patients with psychiatric disorders, mental retardation, or alcohol consumption [1, 2]. Smaller foreign bodies usually pass through the gastrointestinal tract without complications. However, about 10–20% require endoscopic removal, whilst surgery is required in less than 1% for complications such as perforation or ileus [3]. As swallowing is often accompanied by symptoms such as pain or respiratory distress, treatment must be immediate. Only in a few cases there is a documented long-time interval in-between ingestion and onset of symptoms [1, 4, 5]. Ingestion of thermometers is even rarer and according to our knowledge only described in cases with mediastinal or oropharyngeal penetration [4, 6]. Fistulas caused by foreign bodies usually affect the upper aerodigestive tract or the genitourinary system, whereas gastrointestinal fistulas are extremely rare [5, 7]. Ingestion of foreign bodies years or decades ago can lead to the development of fistulas [4, 8].

## Case presentation

A 62-year-old patient presented to the general practitioner with epigastric and upper abdominal pain for 3 days. He was previously known for chronic alcohol consumption and had no history of abdominal operation. The clinical examination revealed a febrile patient (38.0 °C) with an increased heart rate of 132 bpm (beats per minute), a tenderness in the right upper and lower abdomen, coarse rattling sounds in left basal segmental lung as well as increased inflammatory parameters (leucocytosis 20.64 G/l, CRP 234 mg/l). The chest X-ray showed neither pneumonic infiltrates nor free intra-abdominal air, but multiple foreign bodies projected onto the stomach. According to the information provided by the patient he swallowed multiple clinical thermometers 30–40 years ago because of unclear reason. Since this incident he never developed any abdominal pain. The patient was sent to our hospital with suspected ethyl toxic pancreatitis because of increased infection parameters, elevated amylase levels (230 U/l) and stated chronic alcohol consumption.

**Fig. 1:**
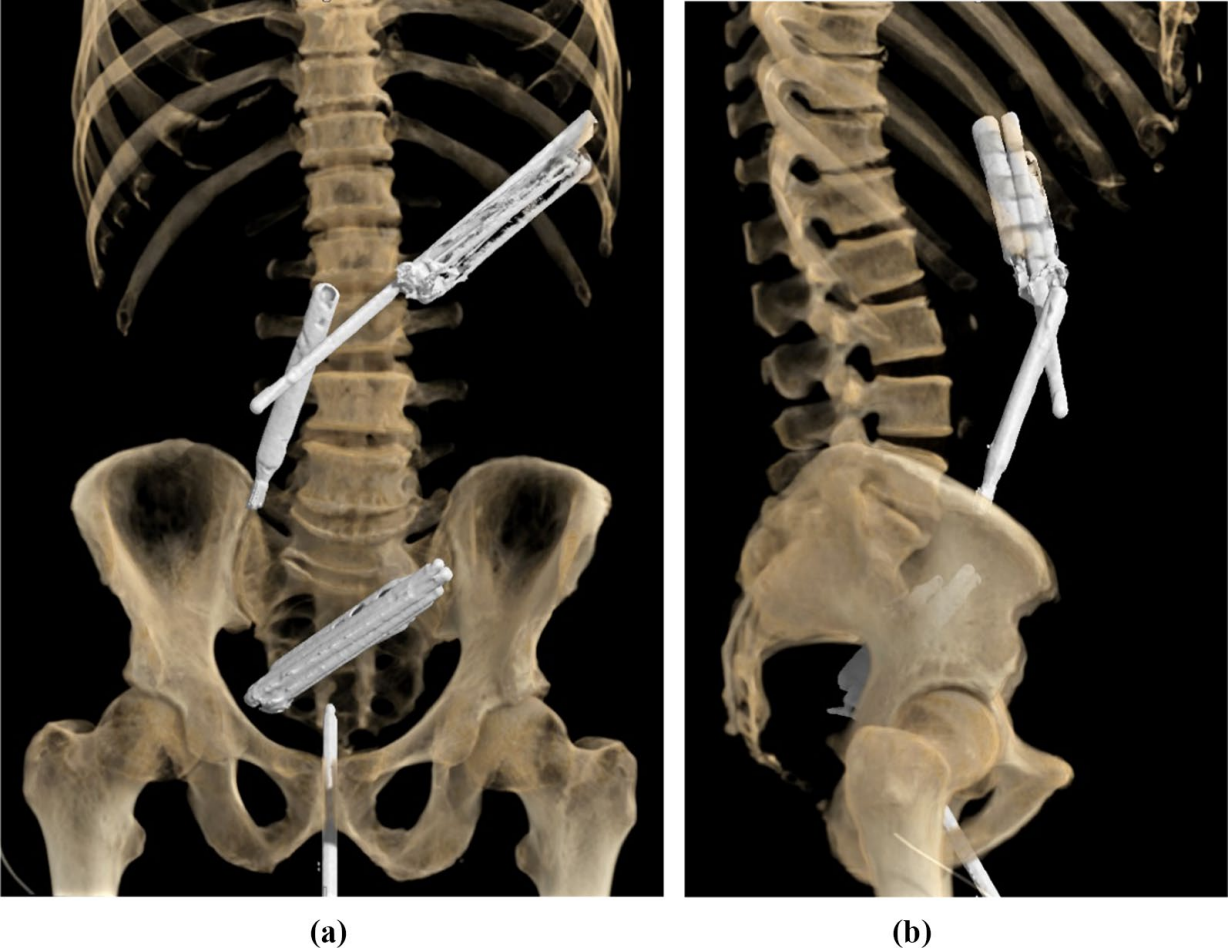
**a**, **b** 3D-reconstruction of CT scan

**Fig. 2:**
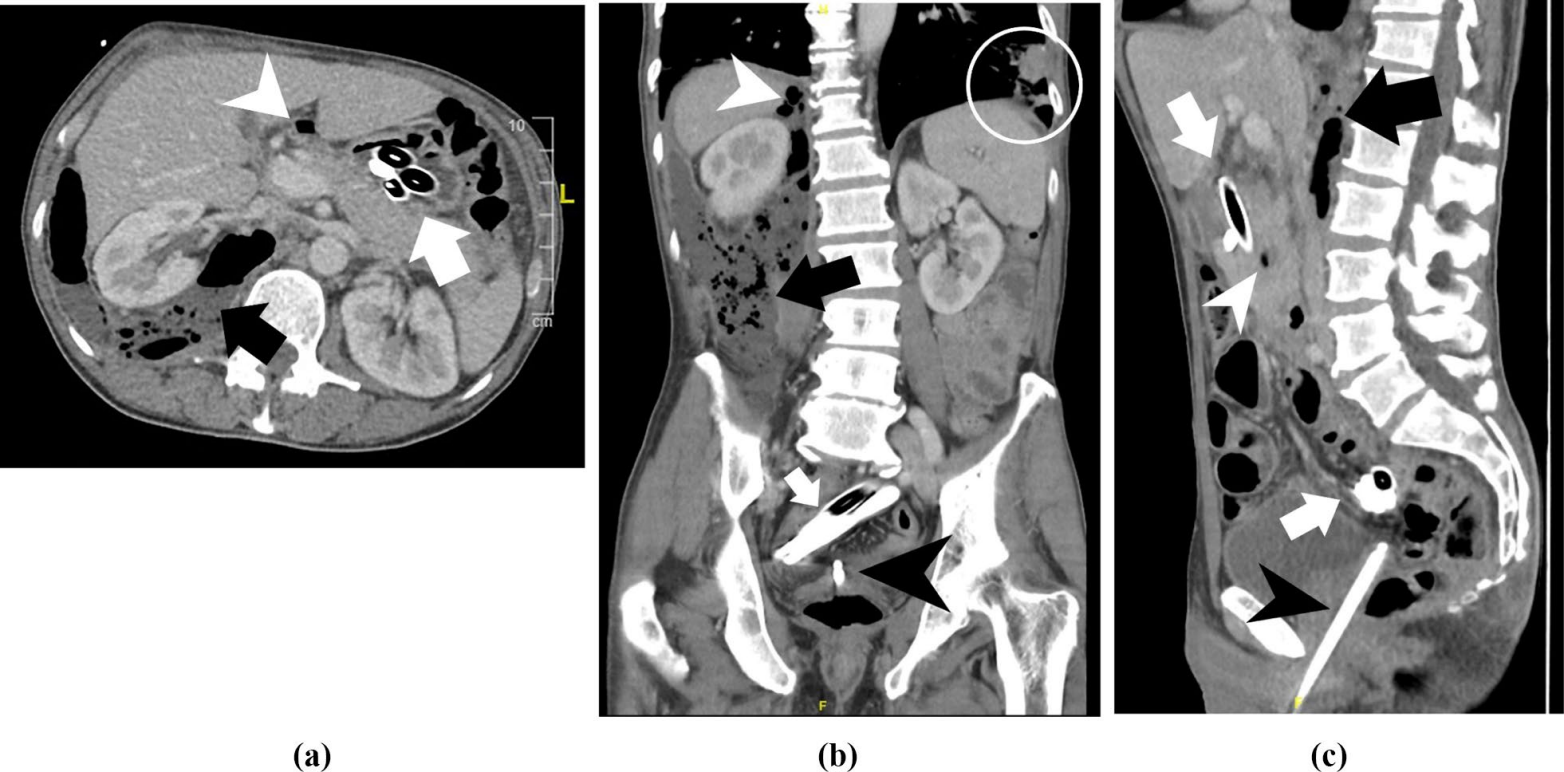
**a**: CT-scan axial - multiple intrabdominal foreign bodies (white arrow), free intra- (white arrowhead) and retroperitoneal air (black arrow), extensive retroperitoneal abscess (black arrow), **b**: CT-scan coronal - multiple intrabdominal foreign bodies (white arrow), free intra- (white arrowhead) and retroperitoneal air (black arrow), extensive retroperitoneal abscess (black arrow), transurethral, intraprostatic foreign body (black arrowhead), pneumonic infiltrate left lower lobe (circle), **c**: CT-scan sagittal - multiple intrabdominal foreign bodies (white arrow), free intra- (white arrowhead) and retroperitoneal air (black arrow), extensive retroperitoneal abscess (black arrow), transscrotal, transurethral, intraprostatic foreign body (black arrowhead)

In our emergency room the patient presented in a poor general and nutritional condition (body mass index 18). Within short time the breathing rate increased to 27/min, non-invasive blood pressure dropped to hypotonic values and Lactate levels were measured at 5.2 mmol/l. We started therapy for septic shock and initiated a computer tomography, as the patient complained increasing abdominal pain. The CT (Computed Tomography) scan showed multiple intrabdominal foreign bodies (Fig. 1a, b), free intra- and retroperitoneal air, and an extensive retroperitoneal abscess because of foreign body-related perforation of pars duodeni I and II, as well as a transscrotal, transurethral and intraprostatic foreign body (Fig. 2a–c). In the left lower lobe of the lung radiologists suspected pneumonic infiltrates.

**Fig. 3:**
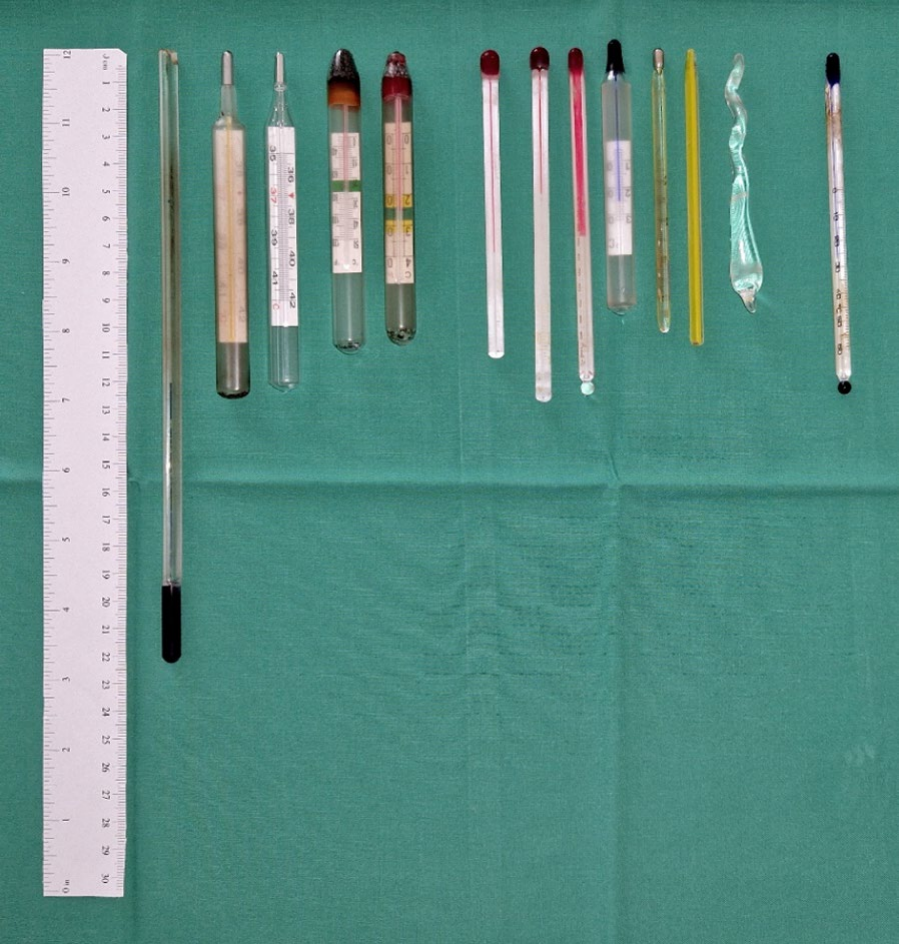
Removed foreign bodies left to right: stomach, ileum, scrotal

**Fig. 4:**
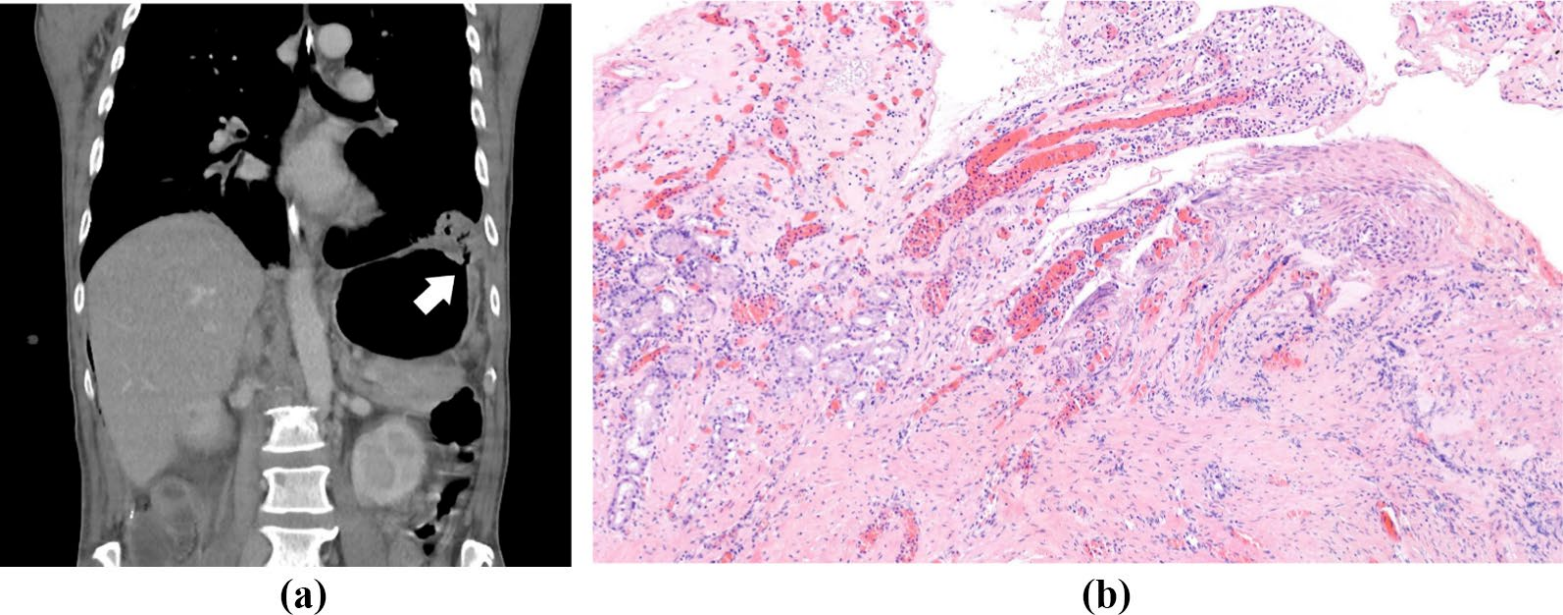
Gastro-thoracal fistula, **a** CT-scan coronal - gastro-thoracal fistula (white arrow); **b** histological specimen

Emergency laparotomy was performed immediately. After removal of the urethroscrotal thermometer by a scrotal incision, gastrotomy was performed first. The gastric and duodenal thermometers were easily removed (Fig. 3) with no evidence of foreign body ingrowth into the hollow organ walls. After lavage and drainage of the retroperitoneal abscess, we removed five thermometers and a glass toy from a pronounced angled loop of the terminal ileum (Fig. 3). Palpation of the gastric fundus appeared inconspicuous. Second look was performed 24 h later approaching the duodenal injuries. Unexpectedly, a duodeno-sigmoid fistula was detected and accordingly excised. Due to inflammatory ischaemic changes of the right colic flexure, right hemicolectomy was performed. Four days later the patient presented a respiratory deterioration. Initiated diagnostic showed a remaining gastro-thoracal fistula (Fig. 4a), which also had to be treated. Six days later the patient suffered from a bursting abdomen, final abdominal wall closure was reached 2 weeks later. During the long hospital stay, the patient got infected to COVID-19 (coronavirus disease of 2019) and was sent to 2-week quarantine the day he was originally planned for discharge to a rehabilitation clinic.

The histological specimen (Fig. 4b) of the gastro-thoracic fistula showed bruised Antral-type gastric mucosa with reactive changes and a 2 mm large considerably contused fragment of squamous mucosa with non-specific chronic, mildly acute inflammation.

We arranged a psychiatric evaluation. The assessment showed signs of congenital mental retardation with credulous behaviour, but no indication of an underlying psychiatric disorder. The patient denied the transurethral insertion of the thermometer, how this thermometer got there remains unclear. The measured Mercury serum level was normal, all thermometers were recovered intact.

## Discussion

The European society of gastrointestinal endoscopy guidelines recommends CT scans when perforation is suspected after ingestion of foreign bodies [5]. As in our case, previous reports also showed that plain radiographs failed to reveal the presence of intra-abdominal free air. CT scans demonstrated the highest sensitivity of imaging techniques in detecting intestinal foreign bodies [9]. In our case CT could not visualize the fistulas, probably because of artefacts caused by the foreign bodies.

**Fig. 5:**
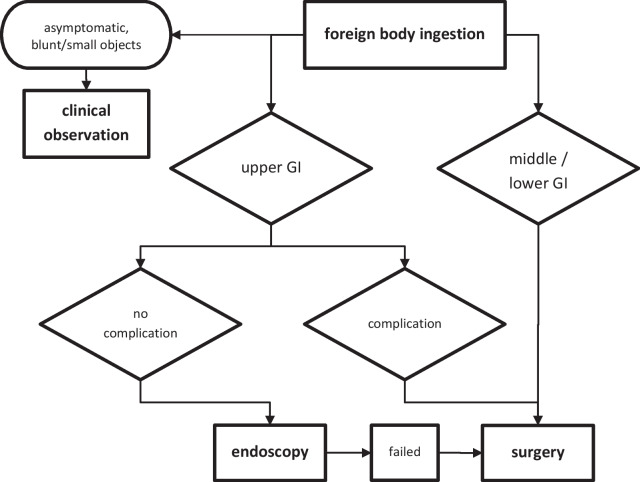
Management of intestinal foreign bodies, recommended by the authors

As shown in this flowchart (Fig. 5) endoscopy is recommended in uncomplicated cases with ingested foreign bodies in the upper GI tract (gastrointestinal tract). If perforation is suspected, endoscopy failed, or other complications occur, surgery should be initiated immediately [10, 11]. Such difficult and unusual cases require a close interdisciplinary communication and collaboration between gastroenterologists and surgeons. Our case confirmed that the foreign bodies were particularly concentrated in areas with angled regions as the duodenum or sigmoid colon and in the area of the most common impaction point orally of the ileocecal valve [9, 12]. Because of the acute course of the disease, we concentrated on the obvious perforation, as ingestion of large foreign bodies is principally associated with perforation of hollow organs. Retrospectively and with our current knowledge, however, a search for fistula must also be carried out as pressure necrosis and inflammation related to the long-foregone ingestion of a foreign body leads to fistula formation. Besides to an intraoperative methylene blue test, further radiological examination using a barium enema could also be carried out in cardiopulmonary stable patients.

## Conclusions

Despite supposedly inconspicuous CT imaging a search for fistulas is necessary, especially in patients with long-foregone foreign body ingestion and a foreign body location in angled regions of the intestinal tract. The search should be performed either intraoperatively or using radiological imaging. In our case with a patient in septic shock a search for fistulas should have taken place during the first operation. Delays due to further radiological examinations would have endangered the patient in this situation but should always be considered in cardiopulmonary stable patients.

### Supplementary Information

**Additional file 1.** Animation 3D-reconstruction of CT-scan.

**Additional file 2.** Animation 3D-reconstruction of CT-scan.

## Data Availability

The dataset supporting the conclusions of this article is included within the article and its additional files.

## Abbreviations

Bpm: Beats per minute
CT: Computed tomography
COVID-19: Coronavirus disease of 2019
GI tract: Gastrointestinal tract
